# Novelty Detection in Underwater Acoustic Environments for Maritime Surveillance Using an Out-of-Distribution Detector for Neural Networks

**DOI:** 10.3390/s26010037

**Published:** 2025-12-20

**Authors:** Nayeon Kim, Minho Kim, Chanil Lee, Chanjun Chun, Hong Kook Kim

**Affiliations:** 1 Department of AI Convergence, Gwangju Institute of Science and Technology, Gwangju 61005, Republic of Korea; nayeunk1117@gm.gist.ac.kr; 2 Underwater Surveillance Technology Research and Development, LIG Nex1, Seongnam-si 13488, Gyeonggi-do, Republic of Korea; minho.kim3@lignex1.com (M.K.); chanil.lee@lignex1.com (C.L.); 3 Department of Computer Engineering, Chosun University, Gwangju 61452, Republic of Korea; cjchun@chosun.ac.kr; 4 Department of Electrical Engineering and Computer Science, Gwangju Institute of Science and Technology, Gwangju 61005, Republic of Korea

**Keywords:** novelty detection, underwater acoustic sensing environments, Monte Carlo dropout, out-of-distribution detector for neural networks (ODIN), DeepShip dataset

## Abstract

Reliable detection of unknown signals is essential for ensuring the robustness of underwater acoustic sensing systems, particularly in maritime security and autonomous navigation. However, Conventional deep learning models often exhibit overconfidence when encountering unknown signals and are unable to quantify predictive uncertainty due to their deterministic inference process. To address these limitations, this study proposes a novelty detection framework that integrates an out-of-distribution detector for neural networks (ODIN) with Monte Carlo (MC) dropout. ODIN mitigates model overconfidence and enhances the separability between known and unknown signals through softmax probability calibration, while MC dropout introduces stochasticity via multiple forward passes to estimate predictive uncertainty—an element critical for stable sensing in real-world underwater environments. The resulting probabilistic outputs are modeled using Gaussian mixture models fitted to ODIN-calibrated softmax distributions of known classes. The Kullback–Leibler divergence is then employed to quantify deviations of test samples from known class behavior. Experimental evaluations on the DeepShip dataset demonstrate that the proposed method achieves, on average, a 9.5% and 5.39% increase in area under the receiver operating characteristic curve, and a 7.82% and 2.63% reduction in false positive rate at 95% true positive rate, compared to the MC dropout and ODIN baseline, respectively. These results confirm that integrating stochastic inference with ODIN significantly enhances the stability and reliability of novelty detection in underwater acoustic environments.

## 1. Introduction

Underwater acoustic sensing is crucial for maritime surveillance, navigation, and reconnaissance, yet the dynamic and unpredictable ocean environment presents significant challenges to accurate sensing and analysis [1,2]. Passive sound navigation and ranging (sonar) is a widely employed technique in such conditions. It functions by receiving naturally emitted underwater sounds without transmitting signals, avoiding environmental disturbances and eliminating the risk of detection associated with active systems [3]. Due to its stealth properties, passive sonar is considered suitable for sensitive underwater applications, particularly military missions that require strict concealment [4].

Various acoustic analysis tasks have been explored in underwater environments, including vessel identification, marine mammal sound detection, acoustic target recognition, and target localization [5,6,7,8,9]. Across these studies, a consistent finding is that underwater acoustic signals exhibit substantial variabilities influenced by factors, such as depth, temperature, surface conditions and ambient biological or geological noise [5,6,7,8,9]. These variabilities cause signals from the same target class to differ significantly under different environmental conditions, making analysis highly challenging [10]. Thus, they become particularly problematic in novelty detection whose goal is to determine whether an incoming signal corresponds to either a known target or an unknown acoustic event. This is because environmental effects can distort known signals to the point that they resemble unknown inputs, thereby blurring the boundary between known and unknown patterns. Consequently, conventional novelty detection approaches often struggle to maintain robust performance in realistic underwater conditions.

To mitigate this limitation, several reconstruction-based novelty detection methods have been developed, showing promise in identifying previously unseen inputs [11]. These methods determine whether an input corresponds to a known or unknown class by measuring its deviation from the training data distribution. A stacked autoencoder was introduced to enable both the detection of unknown inputs and the classification of known categories [12]. However, these techniques still suffer from overlapping reconstruction error distributions between known and unknown samples, leading to unstable decision boundaries for novelty detection. Additionally, distributional shifts caused by environmental variability frequently lead neural network classifiers to assign overly confident predictions to unknown inputs, making it even more difficult to reliably distinguish unfamiliar signals from known classes. The deterministic nature of these models further limits their robustness in such highly variable environments.

Notably, the environmental variability observed in underwater acoustics can be interpreted as a distributional shift: even signals from known targets may deviate significantly from the training distribution due to recording conditions. This property suggests that the novelty detection problem in underwater environments naturally aligns with the framework of out-of-distribution (OOD) detection, which aims to identify inputs that fall outside the distribution learned from known classes. By framing the novelty detection as an OOD detection problem, it becomes possible to leverage an established OOD technique designed to handle distributional shifts and mitigate the overconfidence issue commonly observed in neural network classifiers.

**Figure 1:**
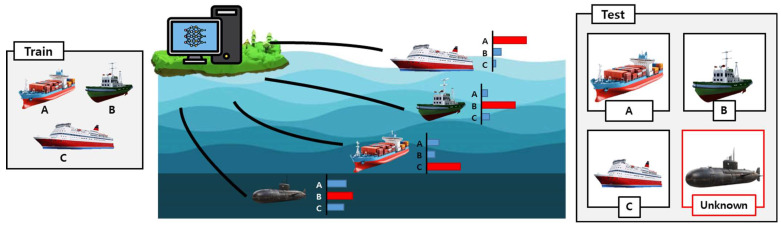
Overview of the novelty detection in the underwater acoustic environment, where A, B, and C denote the known classes used during training. During inference, test samples include instances from classes A, B, C, and unknown. The color bars represent the softmax probabilities of classes A, B, and C for a given instance.

To incorporate this OOD perspective into our study, we first define the datasets for the novelty detection task. Figure 1 illustrates an overview of the novelty detection in the underwater acoustic environment. As shown in the figure, the underwater acoustic dataset consists of multiple vessel classes. The novelty detection task is formulated by withholding one class label during training and treating it as an unknown target during evaluation. A novelty detection model is trained exclusively on the remaining known classes to learn their acoustic characteristics under diverse environmental conditions. During evaluation, the withheld class appears as an unknown acoustic event, requiring the model to distinguish it from the known classes despite potential environmental distortions. This setup reflects realistic underwater sensing scenarios in which previously unobserved vessels or anomalous acoustic sources may emerge, and it provides a structured framework for assessing the model’s ability to detect unknown signals in the OOD framework.

Next, we propose a novelty detection framework for underwater acoustic environments that integrates ODIN and MC dropout to enhance the separability between known and unknown distributions and to overcome the limitations of deterministic models. ODIN mitigates the overconfidence issue by applying temperature scaling and input perturbation, improving the discrimination between known and unknown samples through calibrated softmax probabilities [13]. However, while ODIN effectively reduces overconfidence, it alone is insufficient to capture uncertainty in ambiguous cases. To address this limitation, MC dropout is incorporated, performing multiple stochastic forward passes to estimate predictive uncertainty and generate a calibrated softmax distribution [14]. By combining ODIN’s softmax calibration with MC dropout’s stochastic sampling, the proposed framework is expected to enhance the separability between known and unknown distributions while overcoming the constraints of deterministic prediction. This integration enables more accurate and robust novelty detection in complex underwater acoustic environments.

Building upon the calibrated softmax distributions produced by ODIN and MC dropout, the proposed framework incorporated a distribution-based scoring mechanism that compares the distributions of test samples with class-specific reference distributions. Specifically, a Gaussian mixture model (GMM) is fitted to ODIN-calibrated softmax representations of correctly classified samples from known classes, establishing a statistical baseline for measuring the deviation of test samples from known class distributions. During inference, ODIN and MC dropout are applied to each test sample to generate a sample-specific calibrated softmax distribution, which is subsequently compared with the reference GMM. The KL divergence is used to quantify the discrepancy between the two distributions. The resulting distance is then utilized as a novelty score to determine whether the test sample belongs to a known class or represents an unknown input. The main contributions of this study are as follows:

- This study integrates ODIN and MC dropout for underwater acoustic novelty detection: to the best of our knowledge, this represents the first attempt to combine these two techniques in this domain.
- An uncertainty-aware confidence modeling: we proposed an approach that leverages ODIN-calibrated softmax responses and MC dropout-based stochastic sampling across multiple forward passes to capture predictive uncertainty.
- Improved discrimination under underwater variability: the integrated framework to enhance the discrimination between known and unknown acoustic samples, thereby improving the robustness of underwater acoustic sensing and enabling more reliable, environment-adaptive sensing performance under significant underwater variability.

The remainder of this paper is structured as follows. Section 2 reviews related work on underwater acoustic target recognition and novelty detection techniques. Section 3 presents the proposed framework that integrates ODIN and MC dropout to improve softmax calibration. It describes classifier training, construction of class-wise GMMs, modeling of test sample softmax distributions using ODIN and MC dropout, and computation of novelty scores via a distributional distance metric. Section 4 outlines the dataset, hyperparameter settings, and evaluation metrics. Section 5 reports and analyzes experimental results. Finally, Section 6 concludes the study with future directions for robust underwater acoustic novelty detection.

## 2. Related Works

This study addresses novelty detection in underwater acoustic environments in an OOD detection framework. We review these two areas such as novelty detection and OOD in Section 2.1 and Section 2.2, respectively. This is because existing research works in underwater acoustics have focused primarily on novelty detection, and OOD techniques are largely developed primarily in computer vision.

### 2.1. Novelty Detection in Underwater Acoustic Environments

Novelty detection research for passive sonar in underwater environments has evolved from classical machine learning to deep learning. Early work treated novelty detection as a one-class classification problem, using support vector machines (SVMs) and kernel-based methods to learn decision boundaries that separated known vessel signatures from previously unseen contacts in a high-dimensional feature space [4]. Subsequent instance-based methods exploited similarity between passive sonar signals, using distance- or neighbor-based criteria to compare new observations with stored exemplars, and identified deviations from known acoustic patterns [15].

More recently, deep learning approaches have been introduced. Stacked autoencoders have been trained on signals from known vessels to learn compact latent representations and detect novelties via reconstruction error [2], while hierarchical ensembles of long-short term memory (LSTM)-based autoencoders explicitly modeled the temporal structure of sonar time series at multiple scales to increase sensitivity to anomalous dynamics [3]. In parallel, clustering-and-bagging-based ensembles partitioned the feature space into regions via unsupervised clustering and trained multiple base novelty detectors whose aggregated decisions improved robustness to noise, nonstationarity, and intra-class variability characteristic of underwater acoustic data [11]. Despite these advances, most existing acoustic approaches operate deterministically, necessitating the adoption of probabilistic frameworks to handle the inherent ambiguity of underwater signals.

### 2.2. OOD Detection Techniques

Although OOD detection has received limited attention in underwater acoustics, it has been well established in the computer vision and machine learning domains. Thus, we review the major categories of OOD detection methods: output-based [13,16], gradient-based [17,18], Bayesian [14,19,20,21,22], and distance-based methods [23,24,25,26,27].

OOD data originate from statistically distinct sources, often differing in semantics, sensor characteristics, or environmental conditions, while in-distribution (ID) data comprises samples drawn from the same distribution used during training. Output-based methods, including maximum softmax probability (MSP), ODIN, and generalized ODIN, differentiate between ID and OOD data by applying thresholds to softmax outputs and represent some of the earliest and most widely adopted approaches [13,21]. Subsequently, gradient-based methods were introduced, leveraging the observation that gradient behavior differs between ID and OOD samples, enabling effective detection through variations in gradient direction and magnitude [17,18]. To overcome the limitations of deterministic models, uncertainty-based approaches, such as MC dropout, were introduced [14,22]. These methods estimate predictive uncertainty by activating dropout during inference and conducting multiple stochastic forward passes, improving the separability between ID and OOD samples. Additionally, statistical distance-based methods have been explored, including those employing Mahalanobis distance and probabilistic metrics, such as KL divergence, Jenson-Shannon divergence (JS divergence), and Wasserstein distance. These techniques quantify the deviation of test samples from the ID distribution, providing a more robust basis for OOD detection [16,23,24,25,26,27].

While identifying unknown vessel classes aligns with conventional novelty detection, we formulate it within the OOD detection framework. In addition to OOD, we integrate probabilistic uncertainty estimation based on MC dropout into novelty detection to overcome the limitations of deterministic acoustic novelty detectors.

## 3. Proposed Framework for Novelty Detection in Underwater Acoustic Environments

**Figure 2:**
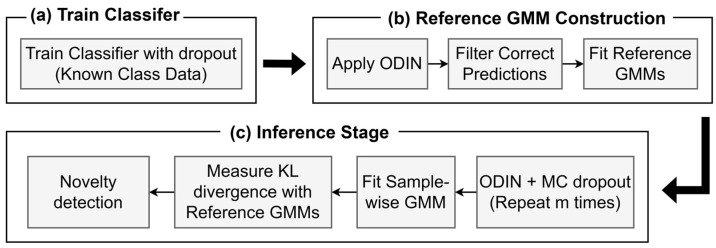
Overview of the proposed novelty detection framework comprising: (**a**) classifier training with dropout; (**b**) construction of class-wise reference softmax probability distributions using ODIN and GMMs, and (**c**) inference-stage novelty detection with ODIN, MC dropout, and distributional distance-based scoring.

This section presents the overall procedure of the proposed novelty detection framework. The framework integrates classifier training, construction of reference GMM using ODIN-calibrated softmax probabilities, and novelty detection during inference based on ODIN and MC dropout. Figure 2 shows an overview of the complete pipeline.

In the classifier training stage, the neural network is trained using only known class data with dropout activated. Subsequently, the class-wise reference GMMs were constructed to serve as statistical representations of the model’s expected softmax probabilities for known class samples. Each GMM captured the variability present in ODIN-calibrated softmax probabilities corresponding to a specific class. This design is motivated by the limitations of single deterministic softmax outputs, which fail to represent class-dependent variance in known class responses and often lead to unreliable novelty detection. The reference distributions thus function as statistical baselines for evaluating test samples, with a separate GMM fitted for each class using correctly classified known samples.

During inference, the framework models a sample-specific softmax probability distribution through multiple stochastic forward passes. The KL divergence is then computed between the sample-wise distribution and each class-wise reference GMM. Based on the minimum divergence value, the test sample is either classified as known or unknown. The detailed procedures are described in the following subsections.

### 3.1. Classifier Training

The first stage of the proposed novelty detection framework involves classifier training, which establishes the basis for later softmax probability distribution modeling and novelty detection. In this phase, a neural network is trained solely on known class data to learn discriminative representations and construct a classifier dedicated to known classes. Dropout layers are embedded within the network architecture to facilitate stochastic forward propagation during inference through MC dropout. The resulting trained classifier outputs the softmax probabilities used to form reference probability distributions and to assess test samples during the detection process.

A Convolutional Neural Network (CNN) architecture named advanced residual network (AResNet) was employed, derived from a modified residual network (ResNet) integrated with a channel attention mechanism, following the underwater acoustic classification model presented in [28,29]. To adapt the architecture for the present application, two major modifications were introduced: (1) the number of input channels was adjusted to accommodate mel-frequency cepstral coefficient (MFCC) inputs instead of the multi-feature fusion used in the original design, and (2) dropout layers were inserted after each attention-based residual blocks (AConvBlocks) to enable stochastic sampling through MC dropout. These modifications retained the core architectural principles of the original model while enabling softmax probability distribution modeling in the subsequent stages.

**Figure 3:**
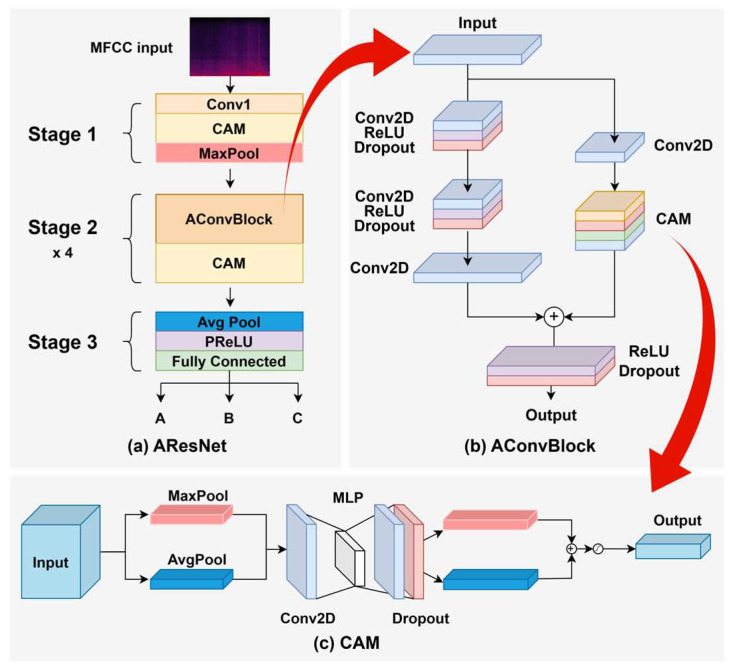
Architecture of the proposed classification model: (**a**) three-stage AResNet architecture, where MFCC inputs are classified into one of the trained classes, A, B, or C, (**b**) structure of the AConvBlock used in Stage 2 of AResNet, and (**c**) detailed design of the CAM module in the AConvBlock.

Figure 3 shows the three-stage model architecture. The first stage performed an initial feature extraction through convolutional layers, followed by batch normalization, rectified linear unit (ReLU) activation, a channel attention module, and max pooling, enabling low-level feature extraction while reducing temporal resolution [30]. The second stage included four AConvBlocks, each integrating residual connections and Channel Attention Modules (CAMs), with progressively increasing channel dimensions and halved temporal dimensions [31]. The third stage applied global average pooling, followed by a parametric ReLU (PReLU) activation function and classification through a fully connected layer [32]. During inference, all dropout layers remained active to perform MC dropout, allowing stochastic predictions for robust novelty detection. The detailed configurations of each layer are presented in Table 1.

For the classifier training stage of the proposed novelty detection framework, the experimental configuration was designed to ensure stable and effective model learning. Cross-entropy loss was used as the objective function, and the Adam optimizer was employed for parameter optimization. Training was performed with a batch size of 16 and a fixed learning rate of 0.0001. To enhance robustness and enable MC dropout during inference, dropout was applied with rates between 0.1 and 0.5. A weight decay coefficient of 0.0001 was incorporated to improve generalization. Additionally, the learning rate was adaptively reduced by a factor of 0.6 every 10 epochs using a step scheduler. These configurations were consistently maintained to ensure a controlled training environment suitable for constructing reliable confidence distributions for novelty detection.

### 3.2. Construction of Reference Distributions

**Figure 4:**
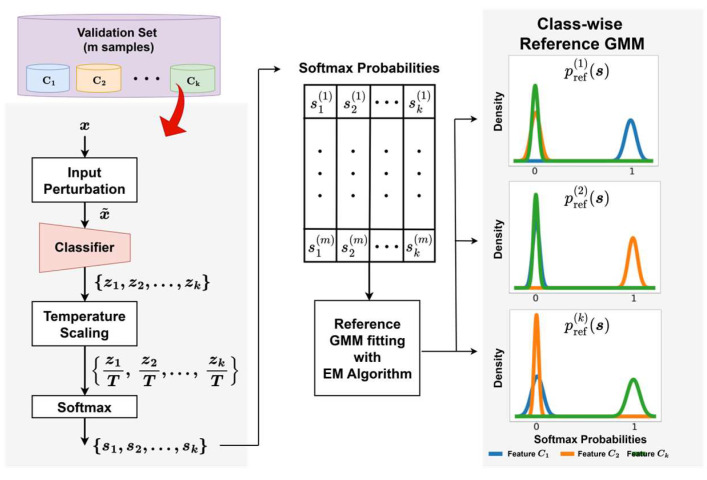
Overview of the reference GMM construction process.

Understanding the distribution of known classes and capturing the subtle variations distinguishing unknown class distributions provides a strong foundation for effective novelty detection. To implement this concept, the proposed framework constructs reference distributions based on calibrated softmax probabilities, which are later used to compare test samples. This section describes the procedure for constructing these distributions using ODIN-calibrated softmax probabilities. The process begins with input perturbation, achieved by computing the gradient of the log softmax of temperature-scaled logits with respect to the input features. This perturbation process was applied to each validation sample x, producing a perturbed input $\tilde{x}$ that was more sensitive to subtle inter-class variations (Figure 4). The perturbed input was then passed through the classifier to generate logits $\left\{z_{1},z_{2},\cdots ,z_{k}\right\}$, followed by temperature scaling in the form $\left\{\frac{z_{1}}{T},\frac{z_{2}}{T},\cdots ,\frac{z_{k}}{T}\right\}$. This temperature scaling reduced overconfident predictions and yielded better-calibrated softmax probabilities. The scaled logits were subsequently processed through a softmax function to produce probability vectors $s=\{s_{1},s_{2},\cdots ,s_{k}\}$.

The obtained softmax vectors were grouped according to their true class labels and used to fit class-wise GMMs through the expectation-maximization (EM) algorithm. The resulting GMMs, denoted as $p_{ref}^{\left(1\right)}\left(s\right),p_{ref}^{\left(2\right)}\left(s\right),\cdots ,p_{ref}^{\left(k\right)}\left(s\right)$ and illustrated on the right side of Figure 3, modeled the distributions of softmax outputs for each known class, effectively capturing the variability in the classifier’s predictions. Representing softmax outputs as probabilistic distributions rather than single-point estimates provided a statistically grounded basis for comparing test samples with the reference distributions. GMMs were particularly suitable for this purpose, as they flexibly model intra-class relationships and covariance structures within the softmax output space. In this study, full covariance matrices were adopted to capture correlations among softmax dimensions, providing a more expressive representation of class-wise uncertainty than diagonal covariance assumptions.

#### 3.2.1. Softmax Calibration Using ODIN

Within the proposed framework, softmax calibration was performed using ODIN, which refined model predictions through input perturbation and temperature scaling. Specifically, input perturbation introduced small, controlled modifications to the input data, increasing the model’s sensitivity to subtle distinctions between known and unknown classes [8]. This adjustment mitigated the overconfidence in the softmax outputs and enhanced the model’s ability to distinguish between known and unknown inputs. The perturbation was applied to the input x as follows:

(1) $$\tilde{x}=x-\epsilon \cdot sign(-\nabla_{x}logS_{T}(y|x))$$

where the perturbation magnitude ϵ serves as a hyperparameter that controls the extent of input modification, determining how strongly the input is altered to improve the separation between known and unknown classes. The perturbation is computed using the gradient of the softmax probabilities with respect to the input, expressed as $\nabla_{x}{logS}_{T}(y|x)$. This gradient represents the sensitivity of the model’s softmax output to small variations in the input. The sign function ensures that the perturbation is applied in the direction that maximally influences the softmax probabilities, enhancing the model’s discriminative capability.

In addition to input perturbation, temperature scaling is applied to mitigate the overconfidence commonly observed in neural network predictions. It modifies the softmax output distribution by flattening the predicted probability values, resulting in better-calibrated probability estimates for both known and unknown classes. This smoothing effect is particularly pronounced for unknown samples, which generally exhibit weaker class evidence, enhancing their separability from known classes in subsequent distributional modeling. The temperature-scaled softmax is given as

(2) $$S_{T}\left(y=k|x\right)=\frac{\exp\left(\frac{z_{k}}{T}\right)}{\sum_{j}exp(\frac{z_{j}}{T})}$$

where $S_{T}(y=k|x)$ represents the probability that the input x is classified as class k under temperature scaling, $z_{k}$ denotes the logit corresponding to class k, and T functions as a scaling parameter that either smooths or sharpens the softmax output. The numerator $exp(z_{k}/T)$ adjusts each logit before exponentiation, while the denominator $\sum_{j}exp(z_{j}/T)$ normalizes the probabilities across all classes. When T=1, the formulation is equivalent to the standard softmax. As T > 1, the resulting probability distribution becomes flatter, suppressing overconfident predictions. This softening effect is particularly beneficial for lowering confidence on unfamiliar inputs, enhancing the model’s ability to distinguish unknown samples from known classes.

#### 3.2.2. Modeling Class-Wise Reference Distributions with a Gaussian Mixture Model

To construct robust reference distributions for novelty detection, we adopt a probabilistic modeling approach grounded in the theoretical connection between softmax classifiers and Gaussian discriminant analysis. A previous work indicated that the deep feature representations learned by softmax classifiers could be effectively approximated using class-conditional Gaussian distributions [16]. Leveraging this insight, we model the ODIN-calibrated softmax probability vectors for each known class using a GMM framework. Unlike the standard linear discriminant analysis assumptions, which enforce a tied covariance matrix across all classes, our formulation employs class-specific full covariance matrices. This design allows the model to capture differences in intra-class variability among vessel classes, which may arise from diverse operating conditions and environmental factors. In this study, we utilize a single Gaussian component (L = 1) per class, effectively reducing the GMM to a multivariate Gaussian model with a full covariance matrix. This configuration is sufficient to capture the unimodal distribution of the calibrated softmax probabilities.

Each GMM is fitted to the softmax probability vectors obtained from correctly classified validation samples, with ODIN calibration applied beforehand to refine the outputs. A distinct GMM is trained for each known class to explicitly capture its unique statistical characteristics. By incorporating the full covariance matrix, the model effectively represents complex geometric relationships among logits, enabling accurate modeling of distributional properties in the high-dimensional softmax space.

The GMM models the probability density of softmax probabilities as a weighted sum of k Gaussian components with a set of m calibrated probabilities vectors $\left\{s^{\left(1\right)},\;s^{\left(2\right)},\cdots ,s^{\left(m\right)}\right\}$, where each $s^{\left(i\right)}\in R^{k}$. The GMM models their probability density as

(3) $$p\left(s\right)=\sum_{j=1}^{L}\pi_{j}N(s|\mu_{j},\;\Sigma_{j})$$

where $N(s|\mu_{j},\Sigma_{j})$ represents a Gaussian distribution characterized by the mixture weight $\pi_{j}$, mean vector $\mu_{j}$, and full covariance matrix of the j-th Gaussian component $\Sigma_{j}$. These parameters are optimized using the expectation-maximization (EM) algorithm, which iteratively refines the model to achieve an optimal fit between the estimated distribution and the observed data.

The EM algorithm consists of two main steps: the E-step and M-step. In the E-step, the responsibility $\gamma_{ij}$ is computed, representing the degree to which the j-th Gaussian component accounts for the sample $s_{i}$ under the current model parameters. In the M-step, the mixture weights $\pi_{j}$, means $\mu_{j}$, and covariance matrices $\Sigma_{j}$ are updated based on these responsibilities. Each parameter is recalculated as a weighted average over all samples, where samples contribute more significantly to components that best describe them. This iterative process enables the model to refine its parameters and better approximate the underlying data distribution. The formal expressions for the EM algorithm are given as:

- E-step: For each softmax probability vector $s_{i}$, the responsibility $\gamma_{ij}$ assigned to the *j*-th component is computed as follows:

(4) $$\gamma_{ij}=\frac{\pi_{j}N\left(s_{i}|\mu_{j},\;\Sigma_{j}\right)}{\sum_{L}\pi_{L}N(s_{i}|\mu_{L,\;}\Sigma_{L})\;\;}.$$

- M-step: The model parameters are updated using the computed responsibilities as follows:

(5) $$\pi_{j}^{new}=\frac{1}{m}\sum_{i=1}^{m}\gamma_{ij},$$

(6) $$\mu_{j}^{new}=\frac{\sum_{i=1}^{m}\gamma_{ij}s_{i}}{\sum_{i=1}^{m}\gamma_{ij}},$$

(7) $$\Sigma_{j}^{new}=\frac{1}{\sum_{i=1}^{m}\gamma_{ij}}\sum_{i=1}^{m}\gamma_{ij}\left(s_{i}-\mu_{j}\right){\left(s_{i}-\mu_{j}\right)}^{⊺}.$$

This iterative process continues until convergence is achieved, typically determined when changes in log-likelihood or parameter values fall below a predefined threshold. Through this refinement, the GMM effectively captures the underlying structure of the softmax probability distributions corresponding to the known classes.

To ensure that the reference GMMs accurately represent the softmax probability distributions of known-class samples, the validation set is utilized without applying MC dropout, assuming the dataset is sufficiently large. This approach enables the construction of compact and robust reference distributions without requiring stochastic sampling.

### 3.3. Inference Stage

**Figure 5:**
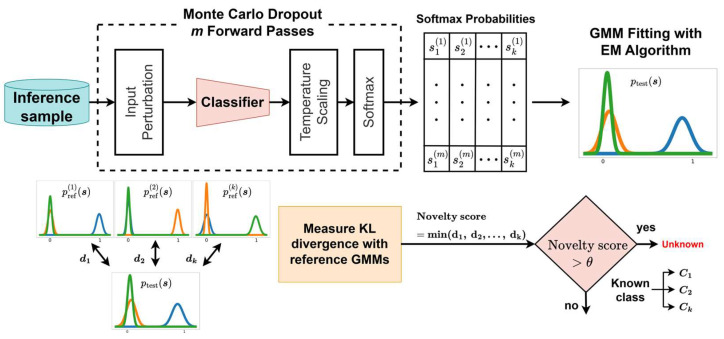
Inference pipeline for novelty detection using ODIN and MC dropout. A test sample is processed through ODIN and MC dropout to generate softmax vectors, which are used to construct a sample-wise GMM. The blue, orange, and green distributions represent the GMMs of logits corresponding to class 1, class 2, class *k*, respectively. The sample-wise GMM is compared with class-wise reference GMMs using the KL divergence, and the minimum divergence is taken as the novelty score for classification.

This subsection presents the inference procedure of the proposed novelty detection framework (Figure 5). For a given test input x, the process begins with input perturbation to emphasize subtle distinctions between known and unknown classes. The perturbed input $\tilde{x}$ is subsequently passed through the classifier multiple times with dropout activated, following the MC dropout strategy. A novelty score is then computed by individually comparing the sample-specific softmax distribution with each of the class-wise reference distributions, allowing the test input to be classified as either known or unknown. The details of GMM modeling during inference and the computation of novelty scores are described in the following subsections.

#### 3.3.1. Modeling GMM at Inference Using ODIN and MC Dropout

This subsection describes the construction of sample-specific confidence distributions during inference using ODIN and MC dropout. First, input perturbation and temperature scaling are applied in the same manner as used for constructing the reference confidence distributions. Subsequently, MC dropout is utilized to produce a set of softmax probability vectors for each test input, capturing stochastic variations arising from multiple forward passes.

Specifically, the perturbed input $\tilde{x}$ is passed through the classifier m times with dropout activated. During each stochastic forward pass, a distinct subset of neurons is randomly deactivated, producing slightly varied outputs [14]. After each pass, temperature scaling is applied to the logits to generate calibrated softmax probabilities, resulting in a collection of softmax probability vectors $\left\{s^{\left(1\right)},\;s^{\left(2\right)},\cdots ,s^{\left(m\right)}\right\}$.

The diverse set of softmax probability vectors obtained through MC dropout is converted into a structured probabilistic representation by fitting a GMM. This GMM models the distribution of the model’s responses to the given test input, effectively capturing its prediction variability. The fitting process follows the same procedure used for constructing the class-wise reference GMMs, ensuring methodological consistency.

Modeling this variability as a distribution over softmax probability vectors enables the GMM to provide a more informative representation than deterministic inference, which produces only a single-point estimate. The resulting distribution captures two essential aspects of the model’s behavior: the average softmax probability across classes and the variability induced by the stochastic nature of dropout. The mean of the softmax probability vectors indicates the most likely predicted class, while the variance reflects the consistency of the model’s predictions across multiple forward passes. Notably, inputs from unknown classes exhibit higher variability, as the model demonstrates increased uncertainty when encountering unfamiliar data. In contrast, known classes display low variance, reflecting the model’s stable and confident predictions for familiar inputs.

This sample-specific GMM therefore serves as a probabilistic representation of the test input’s softmax probability behavior, allowing a robust and statistically grounded comparison with the class-wise reference GMMs during the subsequent novelty scoring stage.

#### 3.3.2. Distributional Comparison for Novelty Detection

Rather than relying solely on point-wise confidence estimates, the proposed method employs distributional comparison. After modeling the sample-specific softmax probability distribution as a GMM, it is compared with the class-wise reference GMMs constructed from correctly classified known-class samples. To quantify the similarity between the test sample and known classes, the KL divergence is used as the statistical distance measure.

KL divergence emphasizes regions where the reference distribution assigns high probability, making it particularly sensitive to subtle deviations in high-density areas [33]. Given two probability distributions, $p_{test}$ and $p_{ref}^{\left(k\right)}$, the KL divergence for class k is defined as

(8) $$d_{k}(p_{test}∥p_{ref}^{\left(k\right)})=\int p_{test}(s)log\frac{p_{test}\left(s\right)}{p_{ref}^{\left(k\right)}\left(s\right)}ds.$$

The resulting KL divergence value serves as the novelty score for the test sample. A higher score indicates a larger deviation from the behavior of known classes, implying that the sample is more likely to belong to an unknown category.

## 4. Experimental Setup

### 4.1. Dataset

In this study, the DeepShip dataset was used to conduct novelty detection experiments in underwater acoustic environments [34]. DeepShip comprises passive sonar recordings collected under various maritime conditions and provides labeled acoustic signals for vessel classification. The acoustic signals were acquired using an IcListen AF hydrophone deployed at a depth of approximately 141–147 m, with an original sampling rate of 32 kHz [34]. The dataset contains four vessel classes representing multiple vessel types and diverse background noise conditions, making it well-suited for supervised learning-based target recognition and novelty detection tasks.

To evaluate the generalizability of the proposed framework across different maritime environments, we additionally utilized the ShipsEar dataset [35]. ShipsEar contains of ship-radiated noise recordings collected from the Spanish Atlantic coast using a digitalHyd SR-1 recorder with a sampling rate of 52,734 Hz. This dataset includes recordings from shallow waters in port environments, offering a distinct acoustic domain with different background noise characteristics distinct from those of DeepShip.

A 10-fold cross-validation procedure was performed. For each fold, the dataset was randomly partitioned into training, validation, and test sets, preserving class proportions. All recordings were resampled at a rate of 8 kHz to maintain consistency across samples, and each audio file—originally varying durations—was segmented into non-overlapping 10 s clips. Table 2 presents an overview of the data partition using the first fold as a representative example, including the number of recording sessions and segmented samples for each vessel class.

For feature extraction, MFCCs are computed to characterize the acoustic properties of the input signals. A total of 60 MFCCs are extracted using 60 Mel filters to partition the frequency spectrum. A 2048-point fast Fourier transform is applied with a window length of 2048 samples to capture temporal variations in the signal. To preserve temporal continuity between frames, a hop length of 512 samples is used, enabling partial frame overlap and improving feature representation.

One of the labeled classes was reserved as the unknown category for evaluation, while the remaining classes were used for model training. The validation set was employed to construct the reference GMM by first applying ODIN calibration and then fitting GMMs to the resulting softmax probabilities. The test set was reserved exclusively for inference and for evaluating the performance of the proposed novelty detection framework.

### 4.2. Hyperparameter Configurations for MC Dropout and ODIN

At the inference stage, MC dropout was applied with varying numbers of stochastic forward passes, specifically 2, 3, 5, 10, 20, 50, and 100. These configurations were selected to evaluate how effectively the variability of unknown samples could be captured and distinguished from the behavior of known classes.

ODIN settings were also varied, with input perturbation magnitudes ϵ set to 0.0001, 0.001, 0.01, and 0.1, and temperature scaling values T set to 1, 5, 10, 50, and 100. These configurations enabled a comparative analysis of different parameter settings. All experimental conditions were applied consistently across tests to ensure a fair, reproducible, and reliable evaluation of the proposed novelty detection framework.

### 4.3. Evaluation Metrics

Performance was evaluated using four metrics: area under the receiver operating characteristic curve (AUROC), false positive rate at 95% true positive rate (FPR@95%TPR), area under the precision-recall curve (AUPR) of ID as positive class (AUPR In), and AUPR of OOD as positive class (AUPR Out) [36,37,38]. These metrics collectively assessed the model’s capability to distinguish between known and unknown classes across varying decision thresholds.

The AUROC metric evaluates novelty detection performance by quantifying how effectively a model distinguishes between known and unknown classes across all possible decision thresholds. It represents the probability that an unknown sample receives a higher novelty score than a known sample. The ROC curve plots the TPR against the FPR, and a larger area under the curve indicates stronger separation capability for unknown classes. An AUROC value near 0.5 corresponds to random performance, whereas a value approaching 1 signifies superior discriminative ability.

FPR at 95% TPR measures the false positive rate when the TPR is fixed at 95%, indicating how frequently known samples are incorrectly classified as unknown while maintaining a high TPR for unknown samples. This metric is essential for assessing a model’s ability to distinguish unknown inputs from known ones without excessive misclassification of ID data. Lower FPR values correspond to better performance, as they indicate fewer misclassified ID samples. FPR is defined as

(9) $$FPR=\frac{FP}{FP+TN}$$

where the false positive (FP) denotes the number of known samples that are incorrectly classified as unknown, whereas the true negative (TN) represents the number of unknown samples that are correctly identified as unknown.

AUPR provides a complementary assessment of detection performance, particularly in scenarios with class imbalance. It is calculated as the area under the precision-recall curve using a discrete approximation. AUPR is evaluated in two forms: AUPR In and AUPR Out. AUPR In treats known data as the positive class and measures the model’s ability to correctly identify known samples while avoiding misclassification of unknown data. In contrast, AUPR Out treats unknown data as the positive class and evaluates the model’s effectiveness in detecting novel or unseen inputs. In both cases, a higher AUPR value indicates greater precision and consistency for the corresponding positive class, reflecting superior detection performance. Unlike AUROC, which evaluates both positive and negative classes equally, AUPR focuses specifically on the performance of a designated positive class. Therefore, in situations where the proportions of known and unknown data are unbalanced, AUPR serves as a more reliable indicator of the model’s precision and recall for the class of interest.

## 5. Performance Analysis

This section presents a detailed analysis of the proposed ODIN+MC dropout framework for novelty detection. Its effectiveness is evaluated by comparing it with established baselines: a hierarchical LSTM autoencoder–based method (LAE-HI), MC dropout, MSP and ODIN, where LAE-HI is used as a reconstruction-based baseline to provide a complementary perspective on novelty detection. MC Dropout is used as an uncertainty-based baseline to isolate the contribution of the proposed distributional scoring mechanism from the benefits of uncertainty estimation alone [14]. It estimates uncertainty by performing multiple stochastic forward passes with dropout activated at a test time and computing confidence scores from the resulting predictive distribution, without applying the proposed GMM-based divergence scoring. MSP serves as a simple baseline that uses the highest softmax output as a confidence score, assuming that unknown samples yield lower maximum probabilities [36]. ODIN improves on MSP by incorporating input perturbation and temperature scaling to compute confidence scores from calibrated softmax probabilities, thereby mitigating the overconfidence commonly observed in neural networks [13]. In the proposed framework, the confidence score is defined as the KL divergence between the test sample’s modeled distribution and its corresponding reference GMM, quantifying the degree of deviation from the known class behavior.

The proposed method, which integrates ODIN with MC dropout and evaluates novelty scores using KL divergence, generally outperforms the LAE-HI, MC dropout, MSP and ODIN baselines across all evaluation metrics (Table 3). Although the magnitude of improvement varies across novelty labels, consistent performance enhancement is observed across all categories. Substantial improvements over all baselines is achieved; for example, the area under the receiver operating characteristic (AUROC) improves by 30.9% relative to LAE-HI. Notably, the method delivers marked improvements in OOD-focused metrics. When Cargo is treated as the unknown class, the area under the precision-recall curve (AUPR) Out increases from 0.2477 to 0.5223, and the false positive rate at 95 true positive rate (FPR@95TPR) decreases by up to 27.16%, indicating a substantially lower false-alarm rate under high-recall operating conditions. This improvement indicates that adopting an OOD detection perspective effectively leverages the inherent variability of underwater acoustic environments, enabling more robust discrimination between known and unknown patterns.

Moreover, the proposed approach outperforms MC dropout by 9.5% and ODIN by 5.39% in AUROC on average, while AUPR Out increases by 11.6% and 7.06%, respectively. This demonstrates that integrating MC dropout with ODIN provides complementary benefits, enhancing the separability between known and unknown signals. Despite the heterogeneous acoustic characteristics across novelty labels, the proposed method consistently improves performance across all metrics, and the lowest AUROC variance across cross-validation folds further highlights its strong generalizability and the stabilizing contribution of MC dropout in reliable novelty detection.

**Figure 6:**
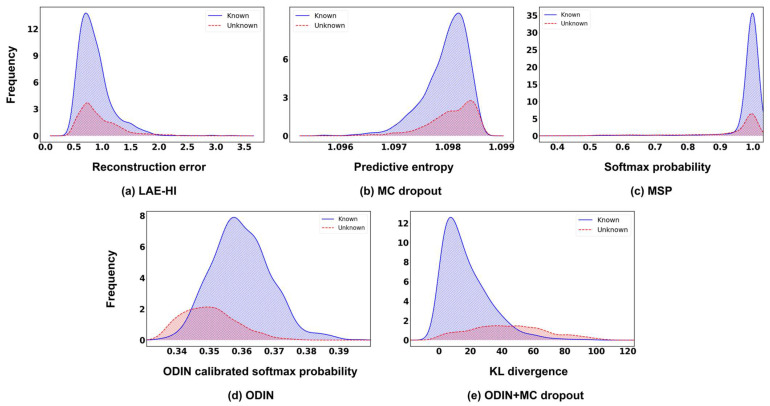
Comparison of confidence score histograms for known and unknown samples with Cargo as the novelty label.

Figure 6 compares the novelty score distributions of known and unknown classes for the baseline methods and the proposed ODIN+MC dropout framework using the DeepShip dataset. In this experiment, ODIN was configured with a temperature of T=100 and a perturbed magnitude of $\epsilon =10^{-2}$. The proposed framework used a dropout rate of 0.3 and 100 stochastic forward passes during inference.

As depicted in Figure 6a, the LAE-HI exhibits substantial overlap between the known and unknown distributions, both forming broad distribution and weakly separated reconstruction-error profiles. This indicates that LAE-HI has a limited ability to capture the structural differences required to distinguish unseen patterns. A similar limitation appears in MC dropout shown in Figure 6b, where it produces uncertainty distributions with widely spread and highly overlapping. Although the unknown samples shift slightly toward higher uncertainty values, the large variance of both distributions makes it difficult to establish a clear and stable threshold based solely on stochastic uncertainty. The MSP method yields confidence score distributions that overlap heavily, with both known and unknown samples concentrated near the upper bound, as indicated in Figure 6c. This indicates excessive model overconfidence and complicates threshold-based discrimination. ODIN mitigates this limitation by calibrating predictions through temperature scaling and input perturbation, thereby improving the separation between known and unknown samples (Figure 6d). However, the two distributions remain similar in overall shape, both exhibiting symmetric bell-shaped curves centered around intermediate confidence values, which limits the effectiveness of threshold-based discrimination.

In contrast, the proposed ODIN+MC dropout method produces confidence score distributions with markedly improved separability. The confidence scores of known classes form a distinct and narrow peak concentrated around low KL divergence values, exhibiting a slightly long-tailed but asymmetric distribution that remains tightly bounded on the lower end. This right-skewed pattern indicates that most known samples are assigned low divergence values with only a few extending into higher regions, which minimizes overlap with the unknown distribution and facilitates a more stable threshold boundary. Conversely, the confidence scores of the unknown class are more broadly distributed and shifted toward higher divergence regions, leading to minimal overlap with the known-class distribution. This pronounced structural distinction between the two distributions facilitates more stable and reliable threshold determination for novelty detection.

**Figure 7:**
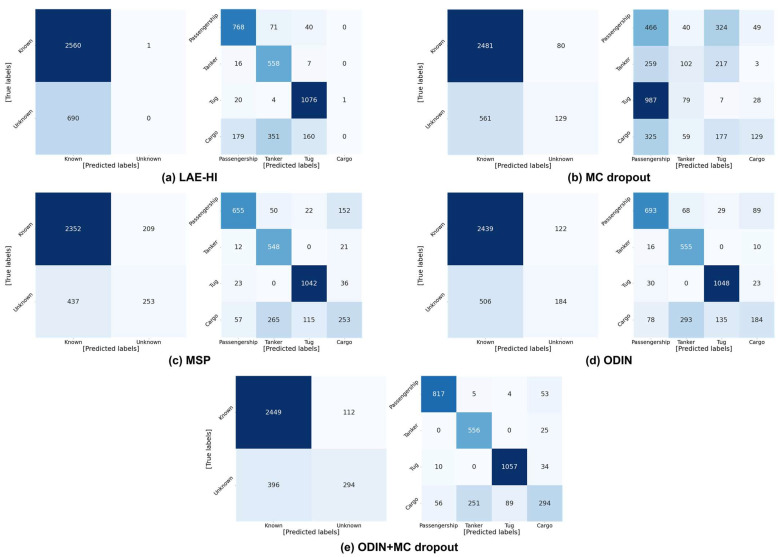
Binary and multi-class confusion matrix across methods: The color intensity reflects the frequency of samples, with darker colors corresponding to higher values.

In real-time novelty detection scenarios, a threshold must be determined to decide whether an input belongs to a known or unknown class. In this work, the threshold was selected at the point where FPR@95TPR is minimized, and novelty detection was evaluated using binary and multi-class confusion matrices. The experimental settings specify the novelty label as Cargo, a temperature scaling factor of 100, and an input perturbation magnitude of 0.01. The resulting binary and multi-class confusion matrices for all evaluated methods are presented in Figure 7, illustrating the comparative performance of each approach under these conditions.

Although LAE-HI provides strong classification performance for known classes, it entirely fails to identify unknown samples. This indicates that the conventional novelty detection approach is ineffective at correctly classifying unknown inputs as unknown. MC dropout offers enhanced binary-detection performance compared to LAE-HI, increasing the number of unknown samples correctly identified unknown samples from 0 to 129. However, its multi-class accuracy degrades from 0.610 to 0.213, due to the influence of predictive entropy, making it less reliable for distinguishing known classes.

MSP, which relies on softmax confidence, achieves a reasonable level of multi-class accuracy (0.768) while improving unknown detection, but it still misclassifies a noticeable proportion of known samples as unknown, resulting in a binary accuracy of 0.801 and limiting its reliability. ODIN achieves slightly higher binary accuracy (0.807) but lower multi-class accuracy (0.763), indicating that it is more conservative in labeling inputs as unknown. Finally, the proposed MC dropout+ODIN approach integrates the strengths of both uncertainty estimation and calibrated softmax responses, achieving the best performance across all metrics. It not only achieves the highest binary accuracy (0.844) and F1 score (0.906) for unknown detection but also maintains strong classification performance for known classes, as evidenced by the multi-class accuracy of 0.838. These results confirm that the proposed method offers the most balanced and robust performance for novelty detection and accurate classification of known classes.

### 5.1. Analysis of ODIN Effect

This subsection examines the influence of ODIN calibration on enhancing novelty detection within the proposed framework. To isolate the effect of each component, the temperature parameter T and perturbation magnitude ϵ are varied independently, and their impact on the separability between known and unknown softmax distributions is analyzed. This ablation study evaluates the individual contributions of temperature scaling and input perturbation to confidence calibration and class separability, as well as their combined effect on the performance of divergence-based novelty scoring.

The distributional characteristics of known and unknown samples are analyzed by visualizing their confidence score histograms under different parameter settings. These comparisons illustrate the individual effects of temperature scaling and input perturbation, as well as the combined impact of both techniques, clarifying their respective and joint contributions to the effectiveness of the proposed novelty detection framework.

#### 5.1.1. Impact of Temperature Scaling on Performance and Distribution

To examine the effect of temperature scaling, the input perturbation magnitude ϵ is fixed at 0 while the temperature scaling factor T is varied. This configuration isolates the influence of temperature on the softmax output distribution and its subsequent impact on novelty detection performance.

**Figure 8:**
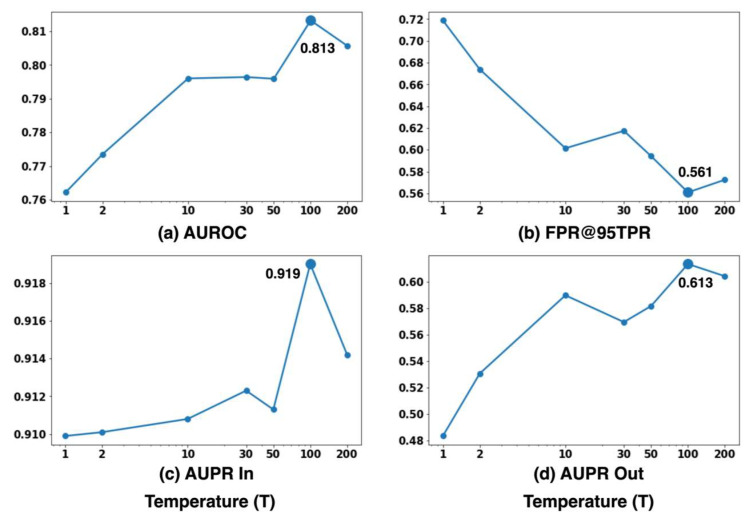
Effect of temperature scaling on novelty detection performance with fixed input perturbation magnitude ϵ=0, and Cargo as the novelty label.

Figure 8 illustrates the effect of temperature scaling on novelty detection performance using the proposed ODIN+MC dropout method. The AUROC value increases consistently with temperature, reaching its maximum of 0.813 at T=100, after which a slight performance decline is observed. Similarly, FPR@95TPR decreases as temperature rises, with the lowest value obtained at T=100, indicating improved reliability in distinguishing between known and unknown samples. The AUPR In also shows a notable improvement at T=100, peaking at 0.919, demonstrating a more favorable precision-recall balance for known-class detection. Lastly, AUPR Out increases with temperature and attains its highest value of 0.613 at T=100 before experiencing a minor reduction.

**Figure 9:**
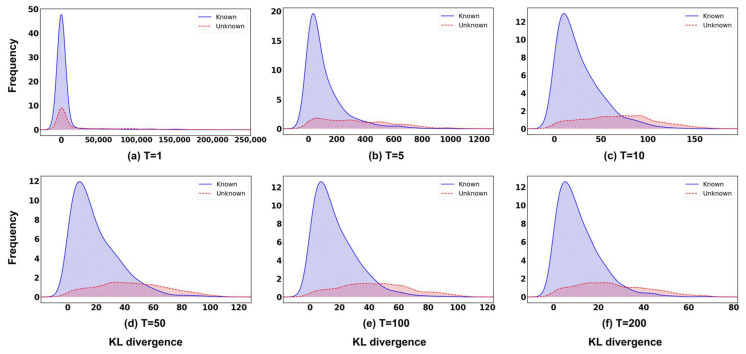
Confidence score histograms obtained using the proposed ODIN+MC dropout method for different temperature values T∈{1,5,10,50,100,200}, with fixed input perturbation magnitude ϵ=0, and Cargo as the novelty class.

Figure 9 illustrate the influence of temperature scaling on the confidence score distributions for known and unknown samples. At lower temperature values, both distributions are densely concentrated near zero, indicating overconfident predictions and poor separability between known and unknown data. As the temperature increases, the confidence scores of known samples shift toward lower divergence values, while the scores of unknown samples become more widely dispersed across a broader range. The most distinct distributional separation is observed at T=100, where the overlap between the two distributions is minimized. Beyond this temperature, the overlap increases once more. These results indicate that moderate temperature scaling effectively mitigates model overconfidence and enhances the contrast between known and unknown confidence patterns, improving novelty detection performance. However, when T becomes excessively large, the separation degrades slightly, likely due to the over-smoothing effect imposed on the softmax outputs.

These findings collectively demonstrate that temperature scaling effectively reduces the overconfidence tendency of neural networks, even in underwater acoustic environments. Specifically, a temperature value around T=100 consistently provides stable and well-calibrated confidence estimation across all evaluation metrics, confirming that ODIN’s temperature scaling mechanism functions as intended within the proposed ODIN+MC dropout framework.

#### 5.1.2. Impact of Perturbation Magnitude on Performance and Distribution

To examine the effect of input perturbation, the temperature scaling factor is fixed at  T=100, while the perturbation magnitude ϵ is varied. This configuration isolates the influence of gradient-based input perturbation on the softmax confidence distributions and its subsequent impact on novelty detection performance.

**Figure 10:**
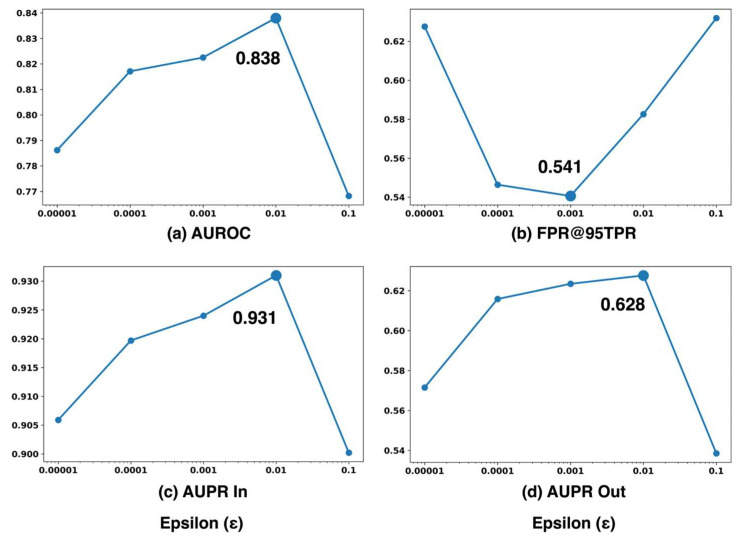
Novelty detection performance for different perturbation magnitudes ϵ with fixed temperature scaling factor T=100 and Cargo as the novelty label.

Figure 10 shows novelty detection performance for different perturbation when Cargo is the novelty label. As shown in the figure, AUROC increases consistently as ϵ rises from $10^{-5}$ to $10^{-2}$, reaching its maximum value of 0.838 at $\epsilon =10^{-2}$ (Figure 10). Beyond this point, performance begins to decline, suggesting that excessively large perturbations introduce unnecessary distortion to the input data. AUPR In and AUPR Out exhibit similar trends, achieving peak values of 0.931 and 0.628, respectively, at $\epsilon =10^{-2}$. These results indicate that a perturbation magnitude of 0.01 provides the optimal balance, enhancing both the separability and calibration of confidence scores for effective novelty detection.

**Figure 11:**
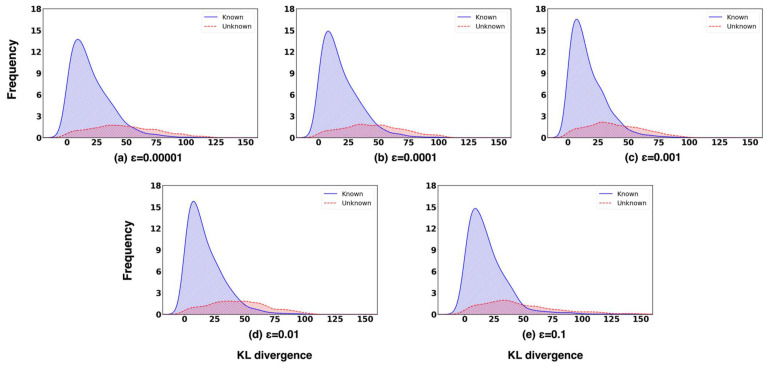
Confidence score histograms of known and unknown samples under different perturbation magnitudes ϵ, with fixed temperature scaling factor T=100 and Cargo as the novelty label.

Figure 11 presents the confidence score distributions of known and unknown classes across different perturbation magnitudes ϵ. Each subplot corresponds to a specific ϵ value, where the distributions of known and unknown samples are depicted in blue and red, respectively. As the ϵ value increases from $10^{-5}$ to $10^{-2}$, the overlap between the confidence score distributions of known and unknown samples gradually decreases, indicating improved separability. At $\epsilon =10^{-2}$, the overlap reaches its minimum, and the region exclusively occupied by unknown samples becomes the widest. This distinct unknown-only region demonstrates that the model assigns clearly differentiated confidence scores to unknown inputs, enabling more reliable and consistent rejection decisions.

The FPR@95TPR exhibits a distinct trend compared to the other evaluation metrics. It decreases steadily as ϵ increases, reaching its minimum value at $\epsilon =10^{-3}$, but begins to rise again once ϵ > 0.01. Interestingly, although performance slightly declines at ϵ=0.01 for this specific metric, the overall improvements observed in AUROC, AUPR In, and AUPR Out compensate for this reduction. These results suggest that small perturbations effectively reduce false positives, but their benefit diminishes when the perturbation strength becomes excessively large.

Overall, it was observed that $\epsilon =10^{-2}$ yielded optimal performance across most evaluation metrics. These results highlight the importance of carefully tuning ϵ to achieve an appropriate balance between robustness and confidence sensitivity within the proposed ODIN+MC dropout framework.

Beyond $\epsilon =10^{-2}$, the overlap between the known and unknown confidence score distributions begins to increase again, while the unknown-only region becomes narrower. This behavior indicates that excessive perturbation distorts input features, diminishing the model’s ability to distinguish unknown samples effectively. Consequently, the confidence scores become less stable, and the separation between known and unknown classes weakens, leading to reduced novelty detection reliability.

These observations confirm that appropriately tuned levels of input perturbation not only reduce distributional overlap but also enhance separability by expanding the region exclusively occupied by unknown samples. This structural improvement within the confidence score space reinforces the effectiveness of the proposed ODIN+MC dropout framework in novelty detection.

### 5.2. Analysis of MC Dropout Effect

The contribution of MC dropout is examined by varying the number of stochastic forward passes M performed during inference, where M∈{2,3,5,10,20,50,100}. For each configuration, ODIN-calibrated softmax outputs are obtained and used to fit a sample-specific GMM, which is subsequently compared with the class-wise reference GMMs to compute divergence-based OOD scores.

To isolate the effect of M on performance, all other parameters are held constant: the temperature scaling factor is fixed at T=100, the input perturbation magnitude at ϵ=0.01, the dropout rate at 0.3, and the novelty class is set to Cargo. Figure 1 illustrates the impact of the number of forward passes M used in MC dropout on novelty detection performance. The parameter  M is varied over {2,3,5,10,20,30,50,100}. AUROC increases sharply as M rises from 2 to 10, reaching its peak value of 0.838 at M=10. Beyond this point, further increases in M result in negligible performance changes, with only minor fluctuations observed. AUPR In follows a similar pattern, stabilizing after achieving its maximum value of 0.9311 at M=20. As shown in the figure, FPR@95TPR decreases steadily as M increases from 2 to 5, reaching its minimum value of 0.567, after which minor oscillations are observed with further increases in M. This trend indicates that a relatively small number of forward passes is generally sufficient to minimize false positives. AUPR Out exhibits a similar pattern, peaking at M=5 and gradually declining thereafter, likely due to over-smoothing effects in confidence distributions when M is large.

**Figure 12:**
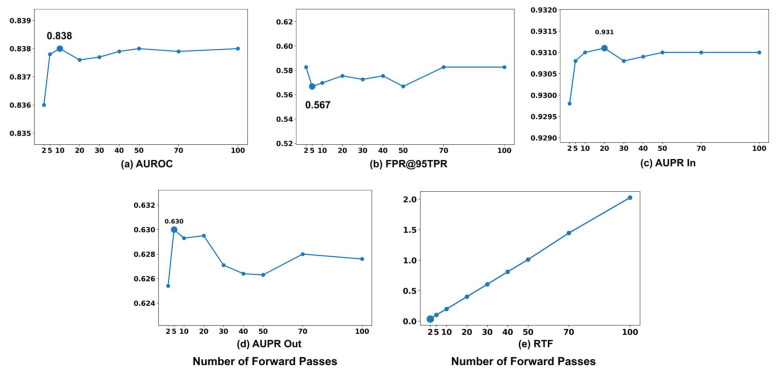
Effect of varying the number of MC dropout forward passes on novelty detection performance.

To explicitly evaluate the performance-complexity trade-off, Figure 12e presents the real time factor (RTF) as a function of M. As clearly illustrated, the RTF exhibits a strict linear increase with the number of stochastic forward passes. While the detection metrics saturate relatively early, the computational cost continues to grow linearly. For instance, increasing M to 100 drastically inflates the RTF to over 2.0 without yielding meaningful performance improvements compared to lower values. Based on this trade-off analysis, selecting a moderate number of forward passes is recommended to achieve robust uncertainty estimation while maintaining a low RTF suitable for practical deployment.

These findings indicate that increasing M improves performance up to a certain threshold, beyond which the benefits plateau. The performance gain from using more than M=20 stochastic samples is marginal across most metrics, suggesting that a moderate number of forward passes is sufficient to capture reliable stochastic features while maintaining computational efficiency. This demonstrates the effectiveness and robustness of the MC dropout-based framework in modeling confidence distributions for reliable novelty detection.

**Figure 13:**
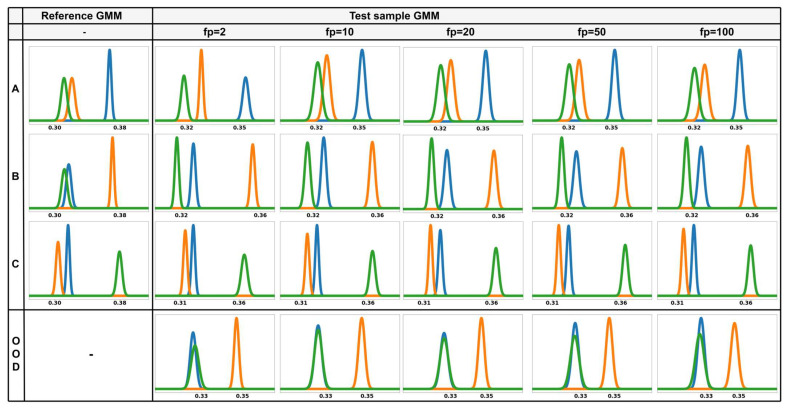
Comparison of GMMs across different numbers of forward passes M, where Reference GMMs and Test GMMs for M={2,10,20,50,100} are shown in the leftmost column and the remaining columns, respectively. The blue, orange, and green distributions represent the GMMs of logits corresponding to classes A, B, and C, respectively.

To examine how the structure of test-time GMMs changes with different numbers of forward passes, a direct comparison is made with the corresponding class-wise reference GMMs. Figure 13 presents these comparison results. The leftmost column displays the reference GMM constructed during training for each class, while the remaining columns illustrate the test-time GMMs obtained by varying the number of forward passes with M∈{2,10,20,50,100}. Each row corresponds to a distinct known class, and each plot represents the ODIN-calibrated confidence score distributions.

When M=2, the test-time GMMs deviate significantly from their corresponding reference structures. The distributions appear misaligned, and the class-wise peaks often display asymmetry or irregular shaping. This discrepancy indicates that with only a few stochastic forward passes, the test GMM lacks sufficient expressiveness and stability to accurately approximate the reference distribution, leading to reduced reliability in confidence estimation.

As M increases, the test-time GMMs progressively align with the structure of the reference GMMs. Beginning at M=20, the peak locations and variances closely match those of the reference distributions, indicating improved fidelity in confidence modeling. This structural convergence enhances the correspondence between the test and reference distributions, allowing for more accurate computation of KL divergence-based novelty scores. However, beyond M=20, the performance improvements become negligible, while computational cost continues to rise, suggesting that excessively large M values are inefficient for practical implementation.

These results demonstrate that MC dropout plays a crucial role in improving the structural alignment between test-time GMM and reference distributions. A sufficiently large number of stochastic forward passes enables the test GMMs to more accurately capture the calibrated confidence patterns learned during training, enhancing the precision of novelty detection. However, as performance gains plateau beyond M=20, selecting an appropriate number of forward passes is essential to achieve an optimal balance between distributional stability and computational efficiency.

### 5.3. Additional Experiments on the ShipsEar Dataset

We conducted an additional evaluation using the ShipsEar dataset to further validate the generalizability and robustness of the proposed framework. The experimental setup, including preprocessing and the One-vs-Rest protocol, remained consistent with the DeepShip experiments.

Table 4 summarizes the novelty detection performance on the ShipsEar dataset. Despite the distributional shift and different in acoustic characteristics from DeepShip, the proposed ODIN+MC dropout framework achieves superior overall performance compared to the baselines. Regarding average AUROC, our method achieves 0.6042, significantly outperforming LAE-HI (0.4930), MC dropout (0.5379), MSP (0.5575), and ODIN (0.5680). Notably, the proposed method consistently improves in AUROC across all four labels.

However, the FPR@95TPR results exhibit class-dependent variability, with ODIN+MC dropout outperforming the baselines for classes A and B, while indicating comparable or higher FPR values for classes C and D. This variation is significantly attributable to the limited dataset size and the inherent imbalance in ShipsEar, which restricts the model’s ability to learn tightly bounded representations for certain classes. Consequently, threshold-based metrics such as FPR@95TPR become more sensitive to class-specific distributional noise, reflecting the dataset’s structural constraints rather than limitations of the proposed framework.

AUPR-Out results show that, although ODIN+MC dropout does not yield the highest score for every individual label and exhibits some label-wise variability, it achieves the highest average score (0.3849) among all methods. This indicates that the proposed framework provides more stable and reliable OOD detection performance when considered across all labels.

Overall, although the ShipsEar dataset’s limited scale and variability introduce performance fluctuations across labels, the consistent improvements in AUROC and the superior average AUPR-Out demonstrate the strength of the proposed method. These results highlight that integrating ODIN with MC dropout provides meaningful robustness gains even in challenging low-resource underwater acoustic environments, reinforcing the method’s practical value and generalization capability.

## 6. Conclusions

This study proposed a novelty detection framework that integrates MC dropout with ODIN to enhance detection performance in underwater acoustic sensing environments. By modeling ODIN-calibrated confidence score distributions with GMMs, the framework enabled divergence-based scoring that quantified structural deviations from known class behavior. The method achieved consistent improvements over LAE-HI, MC dropout, MSP and ODIN baselines across all novelty labels. Compared with LAE-HI, AUROC increased by 30.9%, and further improvements were observed over OOD-detection-based baselines, with the increase of 9.5% over MC dropout, 6.1% over MSP, and 5.4% over ODIN, alongside consistent reductions in FPR@95TPR and increases in both AUPR metrics. These results demonstrated that combining MC dropout with ODIN effectively mitigated overconfidence and distributional variability, thereby enhancing the model’s ability to separate known and unknown signals. By addressing these limitations, the proposed framework achieved more reliable novelty detection in highly variable underwater acoustic sensing environments.

Ablation experiments confirmed that temperature scaling and input perturbation contributed distinctly to confidence calibration, with T=100 and ϵ=0.01 yielding optimal score separability. Increasing the number of MC dropout forward passes improved the stability and alignment of test-time GMMs with reference distributions, although performance gains saturated around M=20, beyond which computational cost outweighed the benefits. Additionally, evaluation on the ShipsEar dataset further demonstrated the generalizability of the proposed framework, indicating consistent improvements across varying acoustic conditions and sensing environments.

While these results indicate promising generalization to a different dataset, they do not guarantee robustness in scenarios where the same known classes appear across datasets but exhibit substantial acoustic variability. Therefore, future work could include evaluation across datasets that share identical known classes but are collected under varying acoustic conditions, allowing assessment of cross-domain consistency and further enhancing the method’s adaptability for practical sensing scenarios.

Future work may also focus on improving distributional separability and reducing computational overhead. Since ODIN combined with uncertainty-based scoring, indicated clear benefits in this study, and ODIN already leverages gradient information through gradient-oriented regularization methods such as GradOrth may further enhance the distinction between known and unknown distributions [18]. Moreover, the increased computational burden imposed by multiple stochastic forward passes limits real-time applicability for continuous underwater monitoring, underscoring the need for more efficient uncertainty estimation strategies suitable for long-term sensing operations.

## Figures and Tables

**Table 1:** Output dimensions of each layer and block in the proposed AResNet model.

Module	Layer/Block Description	Output Shape
Input	MFCC Input (1 channel, 60 frequency bins, 161 time steps)	[−1, 1, 60, 161]
Stage 1	Conv2D (1 × 5, 64 channels)	[−1, 64, 30, 161]
	CAM	[−1, 64, 30, 161]
	Max Pool (2 × 2)	[−1, 64, 15, 81]
Stage 2	AConvBlock 1 (64 → 64) + CAM	[−1, 64, 15, 81]
	AConvBlock 2 (64 → 128) + CAM	[−1, 128, 8, 41]
	AConvBlock 3 (128 → 256) + CAM	[−1, 256, 4, 21]
	AConvBlock 4 (256 → 512) + CAM	[−1, 512, 2, 11]
Stage 3	Average Pool	[−1, 512, 1, 1]
	Fully Connected (512 → 3)	[−1, 3]

**Table 2:** Overview of the DeepShip dataset split for training, validation, and testing. The numbers in parentheses correspond to one representative fold (Fold 1), indicated in the order of training, validation, and test sets.

Class	No. of Files (Train/Val/Test)	No. of Segments (Train/Val/Test)
Cargo	109 (76/21/12)	7671 (5801/1180/690)
Passengership	190 (133/38/19)	11,122 (8177/2066/879)
Tanker	237 (165/47/25)	10,832 (8735/1516/581)
Tug	69 (48/13/8)	7497 (5133/1263/1101)

**Table 3:** Novelty detection performance on the DeepShip dataset evaluated using four evaluation metrics: AUROC, FPR@95TPR, AUPR In, and AUPR Out.

Novelty Label	Method	AUROC (↑)	FPR@95TPR (↓)	AUPR In (↑)	AUPR Out (↑)
Cargo	LAE-HI	0.5334 ± 0.0625	0.9557 ± 0.0322	0.7972 ± 0.0810	0.2477 ± 0.1153
	MC dropout	0.5963 ± 0.3073	0.7469 ± 0.1493	0.8877 ± 0.0628	0.4424 ± 0.1698
	MSP	0.7596 ± 0.0696	0.7381 ± 0.1144	0.9065 ± 0.0496	0.4640 ± 0.1430
	ODIN	0.7600 ± 0.0799	0.7218 ± 0.1140	0.9069 ± 0.0496	0.4836 ± 0.1540
	ODIN+MC dropout	**0.7873 ± 0.0602**	**0.6961 ± 0.1172**	**0.9122 ± 0.0483**	**0.5223 ± 0.1393**
Passengership	LAE-HI	0.5751 ± 0.063	0.8737 ± 0.0546	0.7731 ± 0.1447	0.3367 ± 0.1420
	MC dropout	0.5953 ± 0.2930	0.8901 ± 0.0787	0.7691 ± 0.1387	0.5747 ± 0.2102
	MSP	0.6780 ± 0.0733	0.7990 ± 0.1084	0.7720 ± 0.1246	0.5153 ± 0.2095
	ODIN	0.7018 ± 0.0446	**0.7640 ± 0.1012**	0.7792 ± 0.1272	0.5497 ± 0.2027
	ODIN+MC dropout	**0.7489 ± 0.0205**	0.8184 ± 0.0584	**0.8211 ± 0.1115**	**0.5845 ± 0.1811**
Tanker	LAE-HI	0.5573 ± 0.4567	0.8725 ± 0.3618	0.7780 ± 0.1784	0.2933 ± 0.1752
	MC dropout	0.5411 ± 0.2698	0.8684 ± 0.0164	0.8844 ± 0.0475	0.2789 ± 0.0747
	MSP	0.7006 ± 0.0381	0.8233 ± 0.0682	0.9008 ± 0.0339	0.3361 ± 0.0748
	ODIN	0.6931 ± 0.0456	0.8360 ± 0.0822	0.8977 ± 0.0407	0.3264 ± 0.0714
	ODIN+MC dropout	**0.7179 ± 0.0434**	**0.8221 ± 0.0589**	**0.9068 ± 0.0417**	**0.3426 ± 0.0726**
Tug	LAE-HI	0.6374 ± 0.0742	0.9007 ± 0.0634	0.8342 ± 0.1001	0.3246 ± 0.1454
	MC dropout	0.5693 ± 0.2922	0.7733 ± 0.0912	0.8460 ± 0.1549	0.3856 ± 0.1016
	MSP	0.7228 ± 0.0616	0.8120 ± 0.0571	0.8577 ± 0.1238	0.4356 ± 0.1627
	ODIN	0.7214 ± 0.0766	0.8187 ± 0.0660	0.8564 ± 0.1318	0.4407 ± 0.1410
	ODIN+MC dropout	**0.7511 ± 0.0486**	**0.7355 ± 0.0860**	**0.8825 ± 0.0796**	**0.4815 ± 0.1871**
Average	LAE-HI	0.5730 ± 0.0308	0.8996 ± 0.0177	0.6286 ± 0.0854	0.4736 ± 0.0762
	MC dropout	0.6851 ± 0.0411	0.8361 ± 0.0668	0.7789 ± 0.0996	0.5013 ± 0.1738
	MSP	0.7070 ± 0.0321	0.8057 ± 0.0334	0.7969 ± 0.1023	0.5102 ± 0.1199
	ODIN	0.7118 ± 0.0317	0.7915 ± 0.0524	0.7982 ± 0.0990	0.5224 ± 0.1389
	ODIN+MC dropout	**0.7502 ± 0.0286**	**0.7707 ± 0.0563**	**0.8323 ± 0.0750**	**0.5593 ± 0.1445**

**Table 4:** Novelty detection performance on the ShipsEar dataset was evaluated using four evaluation metrics: AUROC, FPR@95TPR, AUPR In, and AUPR Out.

Novelty Label	Method	AUROC (↑)	FPR@95TPR (↓)	AUPR In (↑)	AUPR Out (↑)
A	LAE-HI	0.4763 ± 0.1983	0.6748 ± 0.2609	0.8609 ± 0.0949	0.1515 ± 0.1594
	MC dropout	0.6472 ± 0.1850	0.6578 ± 0.3256	0.8983 ± 0.0667	0.2359 ± 0.2001
	MSP	0.6664 ± 0.1497	0.6994 ± 0.3403	0.9025 ± 0.0891	0.2308 ± 0.1608
	ODIN	0.7215 ± 0.1014	0.869 ± 0.0924	0.9308 ± 0.0483	0.2601 ± 0.1839
	ODIN+MC dropout	**0.7547 ± 0.1324**	**0.5366 ± 0.2771**	**0.9370 ± 0.0551**	**0.2797 ± 0.2195**
B	LAE-HI	0.5045 ± 0.2432	0.8868 ± 0.1014	0.8419 ± 0.1216	0.1919 ± 0.0878
	MC dropout	0.4652 ± 0.2382	0.7449 ± 0.2107	0.8532 ± 0.1135	0.1548 ± 0.1090
	MSP	0.5129 ± 0.2785	0.8203 ± 0.2626	0.8559 ± 0.1230	0.2305 ± 0.1888
	ODIN	0.5531 ± 0.2634	0.7481 ± 0.3190	0.8693 ± 0.1071	**0.3075 ± 0.2992**
	ODIN+MC dropout	**0.6195 ± 0.0535**	**0.7441 ± 0.0997**	**0.9079 ± 0.0283**	0.2081 ± 0.0611
C	LAE-HI	0.3886 ± 0.1389	0.9588 ± 0.0553	0.5217 ± 0.1921	0.4271 ± 0.1778
	MC dropout	0.5095 ± 0.2114	**0.8566 ± 0.1200**	0.6106 ± 0.2337	0.4731 ± 0.1741
	MSP	0.5408 ± 0.2259	0.8950 ± 0.0824	**0.6651 ± 0.2527**	**0.4854 ± 0.1413**
	ODIN	0.5164 ± 0.1572	0.8804 ± 0.1031	0.6187 ± 0.2214	0.4733 ± 0.1484
	ODIN+MC dropout	**0.5542 ± 0.0566**	0.9089 ± 0.0662	0.6195 ± 0.1946	0.4815 ± 0.1642
D	LAE-HI	0.4664 ± 0.1247	**0.7464 ± 0.1737**	0.7575 ± 0.1757	0.2316 ± 0.1567
	MC dropout	0.4982 ± 0.1049	0.7917 ± 0.1309	**0.7793 ± 0.1533**	0.2526 ± 0.1989
	MSP	0.4659 ± 0.0365	0.9668 ± 0.0447	0.6923 ± 0.1584	0.2998 ± 0.1568
	ODIN	0.5006 ± 0.0730	0.9479 ± 0.0783	0.7150 ± 0.1546	0.3270 ± 0.1826
	ODIN+MC dropout	**0.5199 ± 0.1632**	0.9120 ± 0.0938	0.6900 ± 0.2025	**0.3411 ± 0.1821**
Average	LAE-HI	0.4930 ± 0.0496	0.8482 ± 0.1293	0.6716 ± 0.1559	0.3069 ± 0.1221
	MC dropout	0.5379 ± 0.0804	**0.7871 ± 0.0836**	0.7303 ± 0.1264	0.3510 ± 0.1362
	MSP	0.5575 ± 0.0857	0.8513 ± 0.1142	**0.7452 ± 0.1178**	0.3702 ± 0.1203
	ODIN	0.5680 ± 0.1015	0.8766 ± 0.0832	0.7336 ± 0.1425	0.3825 ± 0.0919
	ODIN+MC dropout	**0.6042 ± 0.1037**	0.8025 ± 0.1775	0.7349 ± 0.1577	**0.3849 ± 0.1161**

## Data Availability

The data presented in this study are openly available in DeepShip at https://github.com/irfankamboh/DeepShip (accessed on 7 November 2025). The data presented in this study are openly available in ShipsEar at https://underwaternoise.atlanttic.uvigo.es (accessed on 2 December 2025).

## Abbreviations

The following abbreviations are used in this manuscript:

ODIN	Out-of-Distribution in Neural Networks
MC	Monte Carlo dropout
GMM	Gaussian mixture model
KL divergence	Kullback–Leibler divergence
SONAR	Sound Navigation and Ranging
OOD	Out-of-Distribution
SVM	Support Vector Machine
LSTM	Long-Short Term Memory
ID	In-Distribution
MSP	Maximum Softmax Probability
JS divergence	Jenson-Shannon
CNN	Convolutional Neural Networks
AResNet	Attention Residual Networks
ResNet	Residual Networks
MFCC	Mel-Frequency Cepstral Coefficient
AConvBlocks	Attention-based residual blocks
ReLU	Rectified Linear Unit
CAM	Channel Attention Module
PReLU	Parametric Rectified Linear Unit
GDA	Gaussian Discriminant Analysis
LDA	Linear Discriminant Analysis
EM	Expectation-Maximization
AUROC	Area Under the Receiver Operating Characteristic Curve
FPR@95TPR	False Positive Rate at 95% True Positive Rate
AUPR	Area Under the Precision-Recall Curve
FP	False Positive
TN	True Negative
RTF	Real-Time Factor
